# An Efficient Classifier for Alzheimer’s Disease Genes Identification

**DOI:** 10.3390/molecules23123140

**Published:** 2018-11-29

**Authors:** Lei Xu, Guangmin Liang, Changrui Liao, Gin-Den Chen, Chi-Chang Chang

**Affiliations:** 1 School of Electronic and Communication Engineering, Shenzhen Polytechnic, Shenzhen 518055, China; csleixu@szpt.edu.cn (L.X.); gmliang@szpt.edu.cn (G.L.); 2 Key Laboratory of Optoelectronic Devices and Systems of Ministry of Education and Guangdong Province, College of Optoelectronic Engineering, Shenzhen University, Shenzhen 518060, China; cliao@szu.edu.cn; 3 Department of Obstetrics and Gynecology, Chung Shan Medical University Hospital, Taichung 40201, Taiwan; 4 School of Medical Informatics, Chung Shan Medical University, Taichung 40201, Taiwan; 5 IT Office, Chung Shan Medical University Hospital, Taichung 40201, Taiwan

**Keywords:** Alzheimer’s disease, gene coding protein, sequence information, support vector machine, classification

## Abstract

Alzheimer’s disease (AD) is considered to one of 10 key diseases leading to death in humans. AD is considered the main cause of brain degeneration, and will lead to dementia. It is beneficial for affected patients to be diagnosed with the disease at an early stage so that efforts to manage the patient can begin as soon as possible. Most existing protocols diagnose AD by way of magnetic resonance imaging (MRI). However, because the size of the images produced is large, existing techniques that employ MRI technology are expensive and time-consuming to perform. With this in mind, in the current study, AD is predicted instead by the use of a support vector machine (SVM) method based on gene-coding protein sequence information. In our proposed method, the frequency of two consecutive amino acids is used to describe the sequence information. The accuracy of the proposed method for identifying AD is 85.7%, which is demonstrated by the obtained experimental results. The experimental results also show that the sequence information of gene-coding proteins can be used to predict AD.

## 1. Introduction

Prior research has shown that there were more than 26.6 million people with AD worldwide in 2010 [1]. It has been predicted that there will soon be a further significant increase in prevalence: specifically, it is expected that there will be 70 million people with AD in 2030 and more than 115 million people with AD in 2050, respectively. In other words, in 2050, one in 85 people are expected to have AD. Unfortunately, to date, there is no treatment in existence that can cure AD. During disease progression, the neurons of AD patients are destroyed gradually, resulting in the loss of cognitive ability and ultimately death. Thus, it is important to identify AD, an age-related disease [2], as early as possible so as to manage the advancement of the condition.

Most existing diagnosis methods focus on identifying AD by way of magnetic resonance imaging (MRI). The MRI method is based on neuroimaging data, for the reason that the imaging data can reflect the structure of brain. Using this technique, the results of classification accuracy are encouraging. However, MRI scans are expensive and the time required for scanning is significant because of the large size of the images. A diffusion map is extended to identify AD in Mattsson [3] and principal component analysis (PCA) is used to reduce features before classification.

Many biomarkers have been discovered for AD identification, such as structural MRI for brain atrophy measurement [4,5,6] functional imaging for hypometabolism quantification [7,8,9], and cerebrospinal fluid for the quantification of specific proteins [6,10,11]. Multimodel data have been employed by multiple biomarkers for identifying AD. Zu et al. [12] predicted AD by using multimodality data to mine the hidden information between features. In Zu [12], the subjects with the same label on a different modal are closer in the selected feature space; as such, a multikernel support vector machine (SVM) can be used to classify the multimodal data, which are represented by the selected features.

As is known, machine learning methods can learn a model from a training sample and then subsequently predict the label of the testing samples. Some machine learning methods have been used to predict AD and mild cognitive impairment (MCI) [13,14,15,16,17,18,19,20]. The information obtained via structural MRI—for example, hippocampal volumes [21,22], cortical thickness [23,24], voxel-wise tissue [23,25,26], and so on—is extracted to classify AD and MCI. Functional imaging, such as fluorodeoxyglucose positron-emission tomography [14,27,28] can also be used for AD and MCI prediction.

Although most existing research has focused on classifying AD based on MRI methods, the cost is expensive. Furthermore, patients often have to have their brain scanned several times in order to inspect the changes in its structure during whole process, increasing the cost even more. Thus, it would be beneficial to find other options for AD identification. Several researches proved that coding genes/noncoding RNAs/proteins were related to diseases, including AD [29,30,31,32,33,34,35,36]. Other investigations [12] have shown that protein structure is related to AD. The gene coding is related to Alzheimer’s disease [37,38,39]. Different from previous work, in the present study, AD is predicted based on protein information. The information of every sequence is represented by a 400-dimension vector, and each dimension represents the frequency of two consecutive amino acids.

**Figure 1:**
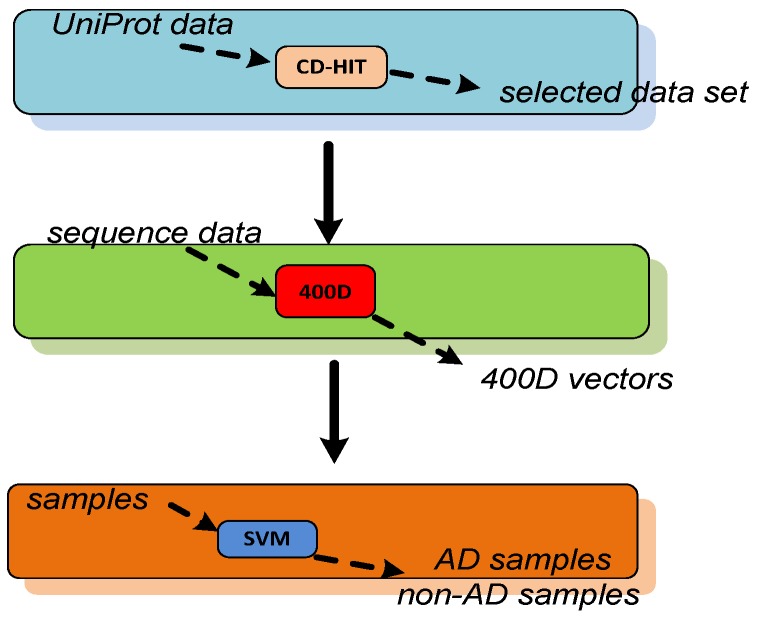
The flow chart of AD identification.

The flow chart of AD identification is shown in Figure 1. First, the data are selected by using the CD-HIT method to remove the most similar sequences. In this step, the input are the proteins related with AD, and the output are selected proteins. Second, the features are extracted from the selected sequences. Each sequence is represented by a 400-dimension (400D) vector. In the third step, the data are classified by a support vector machine method. The input are the feature vectors, and the output are peptides with labels. To the best of our knowledge, this study represents the first effort to identify AD by protein sequence information without the use of MRI. Moreover, a dataset including AD and non-AD samples was created in this work. The experimental results show that the classification accuracy for AD prediction is 85.7%. The contributions of our work include:

(1) A method for predicting AD is proposed in this work. The experimental results demonstrate that the classification accuracy of the proposed method is 85.7%.
(2) Our method is based on protein sequence information. The frequencies of two consecutive amino acids are extracted from the sequence with a 400-dimension vector.
(3) A dataset with AD and non-AD samples is created. This dataset could also be used for additional AD prediction studies.

The rest of the paper is organized as follows: Section 2 introduces the experimental results of the proposed method. The dataset and the proposed method are introduced in Section 3. Finally, the conclusion is made in Section 4.

## 2. Results and Discussion

### 2.1. Results

Identifying AD by way of using protein sequence information has not been widely done yet. Moreover, most existing works use AD Neuroimaging Initiative (ADNI) database [40], which is based on MRI. Existing methods also use MRI information for classification, which is different from our method. Thus, it is difficult to compare the performance evaluation of our proposed method with the performance of existing methods. The performance of our method is shown in Table 1.

As noted in the table, the method was evaluated according to accuracy, precision, recall, F-measure, Mathew coefficient (MCC), and receiver operating characteristic (ROC). The accuracy of the proposed method was 85.7%, which means that the more than 85% of AD and non-AD samples were able to be classified correctly using the method in question. F-measure is based on precision and recall. The recall of our method was 0.857, and the result shows that 85.7% of AD samples in the dataset could be identified in the experiment. Area under the curve (AUC) is related to the metrics of receiver operating characteristic (ROC). ROC is used to measure sensitivity and specificity, while AUC describes the area under the ROC curve. When the AUC is larger, the performance of the algorithm is better. The value of AUC for our method was 0.857 according to the UniProt dataset [41]. The experimental results show that the performance quality of our method in terms of accuracy, precision, and four other metrics as well as the results obtained are acceptable and encouraging.

### 2.2. The Comparison of Performance Evaluation on Feature Selection Methods

**Figure 2:**
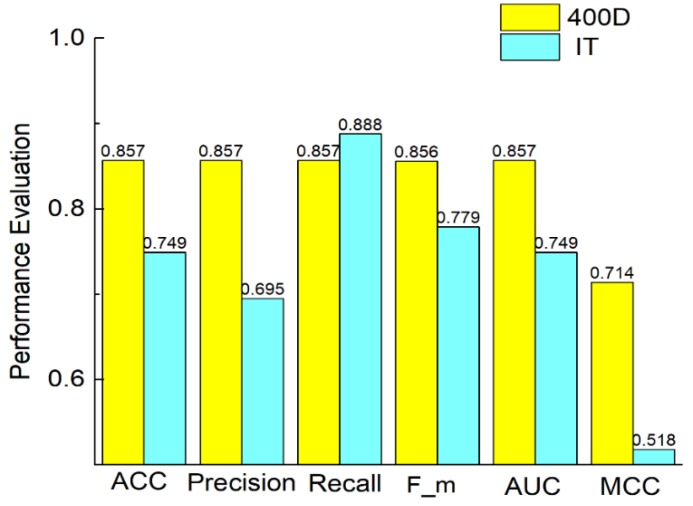
Comparison of 400D with information theory on SVM.

To demonstrate the efficiency of the feature extraction method we used, we compared the 400D features with information theory, which is another feature extraction method. Information theory is proposed in Wei [42], for exploring sequential information from multiple perspectives. Figure 2 shows that 400D performs better than information theory method on accuracy, precision, F-measure, AUC and MCC. The value of recall is higher by using information theory method than using 400D.

### 2.3. The Comparison of Performance Evaluation on Existing Classification Methods

Our method’s performance is evaluated according to other classifiers, such as random forest, naïve Bayes, LibD3C, Adaptive Boosting (AdaBoost), and Bayes network. The classifiers are introduced briefly as follows:

- Random forest is an ensemble classifier, which learns more than one decision tree together. The decision will be made by voting process.
- Naïve Bayes assumes the features are independent of one other. The samples will be assigned to a class with the maximum posterior probability.
- LibD3C [43] is a hybrid ensemble model, which is based on k-means clustering and the framework of dynamic selection and circulating in combination with a sequential search method.
- AdaBoost can assemble classifiers together and, during the training process, the weights of the samples which are classified incorrectly will be increased. The weights of the samples classified correctly will be decreased.
- Bayes network is a probabilistic graph model. The variables and their relationships are represented by a directed acyclic graph.

**Figure 3:**
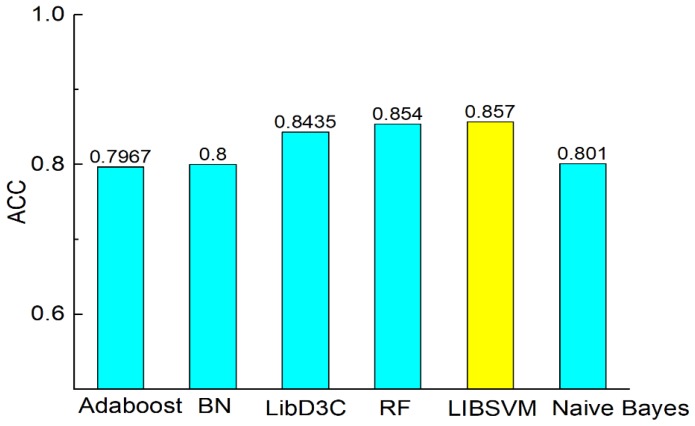
Comparison of ACC on different classifiers.

**Figure 4:**
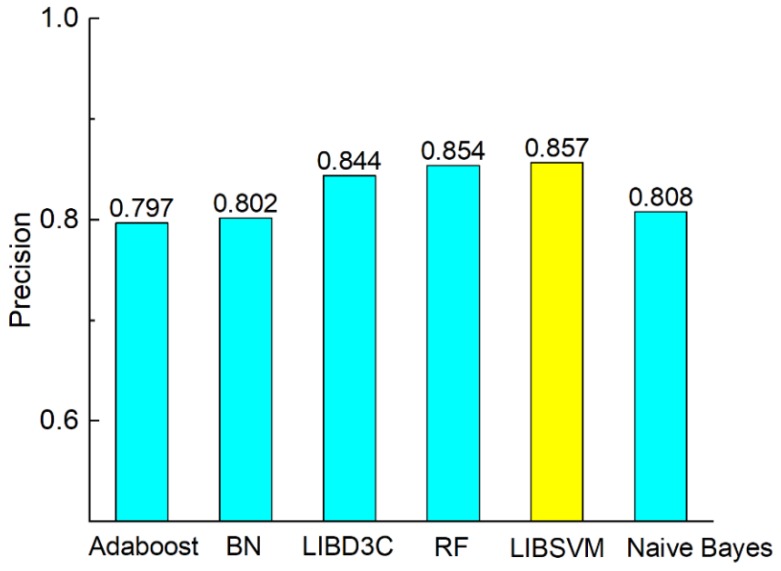
Comparison of precision on different classifiers.

**Figure 5:**
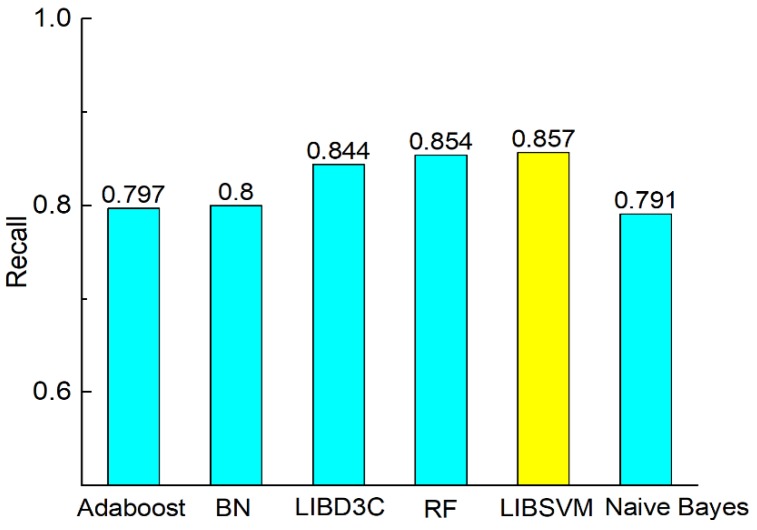
Comparison of recall on different classifiers.

**Figure 6:**
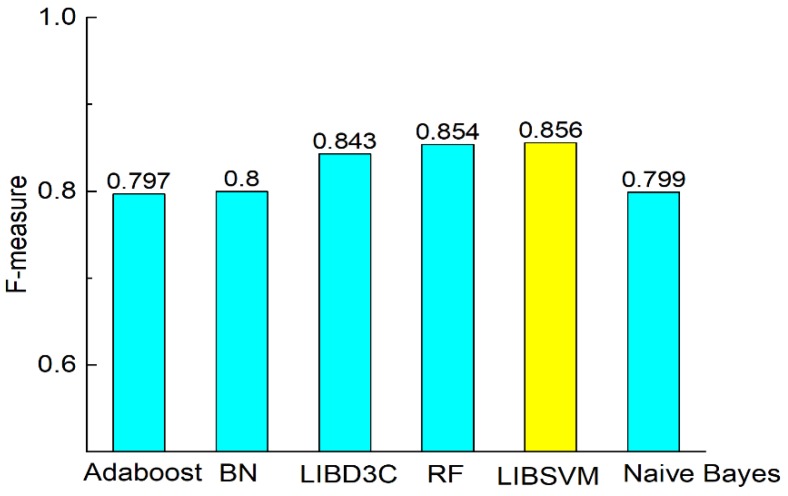
Comparison of F-measure on different classifiers.

**Figure 7:**
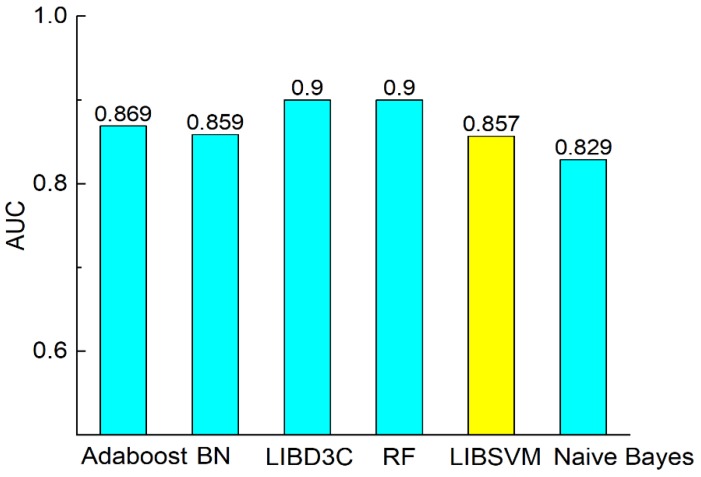
Comparison of AUC on different classifiers.

Figure 3 shows the comparison of accuracy according to the six classifiers. The comparisons of precision, recall, F-measure, MCC, and AUC are shown in Figure 3, Figure 4, Figure 5, Figure 6 and Figure 7. In Figure 3, we can see that accuracy performs better than the other classifiers. The value of accuracy of AdaBoost, Bayes network, and naïve Bayes is about 0.8, while the accuracy of SVM is 0.857. The accuracy of LibD3C is 0.84. The accuracy of random forest is 0.85, which is comparative with that of SVM. Thus, SVM improves the accuracy of other classifiers by nearly 1% to 7%.

**Figure 8:**
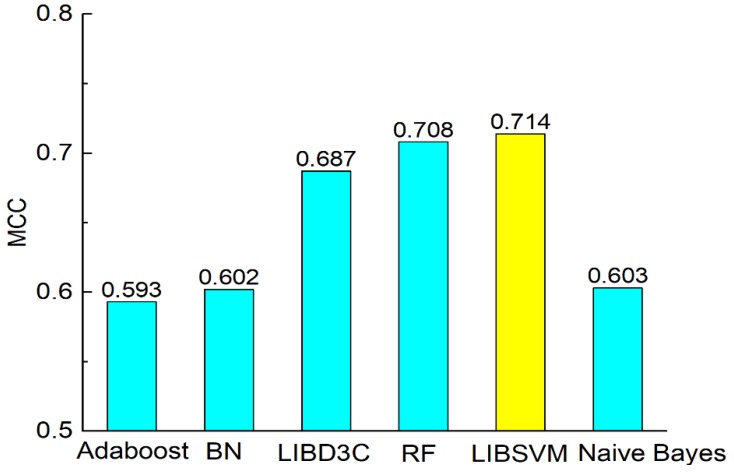
Comparison of MCC on different classifiers.

Figure 4, Figure 5, Figure 6 and Figure 7 show the comparisons of the classifiers on precision, recall, and F-measure. The results are similar to those of Figure 3. SVM performs better than the other methods. The performance is improved by SVM by approximately 1% to 7.5% as compared with in the case of the other methods. F-measure is calculated based on precision and recall, so the result here is consistent with that of precision and recall. AUC reflects the area under the ROC curve. AUC refers to the ratio of the specificity and sensitivity. The value of AUC on random forest is 0.93, which is better than the values achieved via other methods. The values of AUC for AdaBoost, Bayes network, SVM, and naïve Bayes are similar to one another. Figure 8 shows that the MCC of SVM is 0.714, which is better than the MCCs of the other mentioned methods. The values of MCC for random forest and SVM reach a level of 0.7. Moreover, the value of MCC is improved by 0.8% to 20% by using SVM. As a result, SVM performs better than other classifiers evaluated by the metrics.

## 3. Materials and Methods

### 3.1. Benchmark Dataset

The data were selected from the UniProt database [41,44]. To guarantee the validity of the dataset, the proteins with ambiguous meanings (such as “B”, “X”, and so on) is removed, and only the proteins related to “Alzheimer’s disease” are kept.

The benchmark dataset (D) is represented by a positive subset (D^+^) and a negative subset (D^−^), formulated as seen in Equation (1):

(1) $$D=D^{+}\cup D^{-}$$

where the symbol “∪” represents the union of the sets in the set theory. After the selection process, there are 310 proteins related to AD and 312 non-AD proteins left in the benchmark dataset. Because some sequences are significantly similar, the redundancy of the sequences is considered. To avoid the overestimation of the performance of the methods, the homologous sequences with more than 60% similarity were removed from the dataset by using CD-HIT program [45]. As a result, a benchmark dataset with 279 proteins related to AD and 1,463 proteins not related to AD was used for the prediction model. In other words, the benchmark dataset contains 279 positive samples in the positive subset (D^+^) and 1,463 negative samples in the negative subset (D^−^), respectively.

### 3.2. Support Vector Machine

SVM is a supervised machine learning model. The labeled samples are trained based on the goal of maximizing the margin between the classes. Since SVM performs better than some the-state-of-art supervised learning methods, SVM is widely used in classification problems. Most works in bioinformatics [41,46,47,48,49,50,51,52,53,54,55,56,57,58,59] also use SVM for classification. SVM was used to identify AD in our work.

The principles of SVM were introduced in Chou and Cai [60,61], and more details are provided in Cristianini [62]. Above all, the key idea of SVM is that two groups are separated with a maximum margin by building a hyperplane. The objective function of SVM is described in Equation (2), as follows:

(2) $$\underset{w,b}{argmax}\{\frac{1}{\left\|w\right\|}\underset{i=1,2,\ldots ,n}{\min}[y_{i}\left(w^{T}\varphi \left(x^{\left(i\right)}\right)+b\right)]\}$$

In Equation (2), the input variable *x*^(*i*)^ is mapped into a high dimensional feature space by the kernel function φ(·). Radial kernel function (RBF) is used in the experiment. RBF is used widely because of its effectiveness and efficiency. Equation (2) can be transferred to optimize Equation (3), as follows:

(3) $$\max\frac{1}{\left\|w\right\|},\;s.t.\;y_{i}\left(w^{T}\varphi \left(x^{i}\right)+b\right)\geq 1,i=1,\ldots ,n$$

where *n* is the number of training samples. The condition ($y_{i}\left(w^{T}\varphi \left(x^{i}\right)+b\right)\geq 1$) should be satisfied in Equation (3), which means that the samples must be classified correctly by the optimized hyperplane. However, the problem of overfitting will be caused. Soft SVM is proposed to tackle the problem. The objective function is refined into Equation (4), as follows:

(4) $$\begin{array}{c} \underset{w,b}{\min}\left(\frac{1}{2}{\left\|w\right\|}^{2}+C\overset{n}{\underset{i=1}{\sum }}\delta_{i}\right)\\ s.t.\;y_{i}\left(w^{T}\varphi \left(x^{i}\right)+b\right)\geq 1-\delta_{i},i=1,\ldots ,n\\ \delta_{i}\geq 0 \end{array}$$

where $\delta_{i}$ is the slack variable and *C* is the penalty parameter. The SVM used in our work is the package named LIBSVM written by Chang and Lin [63].

### 3.3. Sequence Representation

AD is classified based on protein sequence information, so, in this paper, we used the features extracted from the peptides. The sequence is represented by a 400-dimension vector, and each dimension describes the frequency of two consecutive amino acids. The feature extraction will be introduced later. To describe the information more clearly, the symbols used in the paper are summarized in Table 2.

P_L_ is a peptide with L residue, so P_L_ can be written into a sequence as {R_1_R_2_R_3_…R_i_…R_L_. R_i_ represents the i-th residual of P_L_ in the sequence. The symbol *f_i_* represents the normalized occurrence frequency of the i-th type of native amino acid in the peptide. There are, in total, 20 types of native amino acids. The peptide P can be represented by F_p_ = [*f*_1_, …, *f_i_*, …, *f*_20_], reflecting the occurrence frequency of every amino acid of P. It is obvious that the sequence information is lost in F_p_. To overcome this limitation, we extracted the occurrence frequency of the combination of two consecutive amino acids, such as AR (A and R representing the amino acids). Since there are 20 native amino acids, the number of features of the combination of two consecutive amino acids is 400 (20^2^). Thus, we call it a 400D sequence-based feature. The peptide P is straightly represented by (*f_AA_*, *f_AR_*, …, *f_VV_*).

### 3.4. Performance Evaluation

The classification quality is evaluated by accuracy, recall, precision, F-measure, MCC, and AUC. The metrics are used in evaluating the performance frequently [64,65,66,67,68,69,70,71,72]. In the experiments, n is the number of samples, so *n*^+^ is the number of positive samples and *n*^−^ is the number of negative samples. TP (true positive) represents the number of samples that are labeled positive by the method correctly. FP (false positive) is the number of samples that are labeled positive but which are in fact negative. TN (true negative) means the number of sample which are classified correctly as negative sample. FN (false negative) is the number of samples that are positive but which are labeled as negative. The accuracy (ACC_G_) represents the correct classification rate of a method G, which is shown in Equation (5). Precision_G_, recall_G_ and F-measure_G_ are calculated in Equations (5) through (8). AUC is the area size of the ROC curve. The X-axis of ROC curve is the false positive rate, while the Y-axis is true positive rate. The MCC describes the rate of specificity and sensitivity, which is calculated by Equation (9). Specificity and sensitivity are used in evaluating the performance of protein prediction, such as in the case of Feng [47,48] and so on. Specificity (Sp, calculated by Equation (10)) is the rate of misclassification of AD proteins. Sensitivity (Sn, calculated by Equation (11)) is the rate of correctly classified AD proteins:

(5) $$ACC_{G}=\frac{TP+TN}{n^{+}+n^{-}}$$

(6) $$Precision_{G}=\frac{TP}{TP+FP}$$

(7) $$Recall_{G}=\frac{TP}{TP+FN}$$

(8) $$F-measure_{G}=\frac{\left(1+b^{2}\right)\times P\times R}{b^{2}\times P+R}$$

(9) $$MCC=\frac{Sp}{Sn}$$

(10) $$Sp=\frac{TN}{TN+FP}$$

(11) $$Sn=\frac{TP}{TP+FN}$$

## 4. Conclusions

In this paper, a computational method based on protein sequence information was introduced to predict the onset of AD. In our proposed method, the sequences are represented by the frequency of two consecutive amino acids, and then the data are classified by SVM. Our work is different from previous work that was completed using MRI, which is time-consuming and expensive. As demonstrated by the presented experimental results, the classification accuracy of our proposed method is 85.7%. Moreover, a dataset used for AD classification was created in our work. In future work, we will try to mine the relationships between the features to improve the classification performance of the predictions method. Furthermore, due to the wide use of webservers in bioinformatics, such as the work of RNA secondary structure comparison [73], we will also develop the a webserver for AD prediction.

## Figures and Tables

**Table 1:** The performance of our proposed method.

Performance Evaluation	Accuracy
ACC	0.8565
Precision	0.857
Recall	0.857
F-measure	0.856
MCC	0.714
AUC	0.857

**Table 2:** The symbols used in the present paper.

Symbol	Meaning
P_L_	Peptide with L residual
R_i_	The i-th residual
f_i_	The frequency of the i-th amino acid
F_p_	The feature vector of peptide P
